# Radioprotective effects of vitamin A against gamma radiation in mouse bone marrow cells

**DOI:** 10.1016/j.mex.2019.03.020

**Published:** 2019-04-03

**Authors:** Vahid Changizi, Seyed Abolghasem Haeri, Sakineh Abbasi, Zahra Rajabi, Mohammad Mirdoraghi

**Affiliations:** a Department of Radiology and Radiotherapy Technology, School of Allied Health Sciences, Tehran University of Medical Sciences, Tehran, Iran; b Nuclear Science and Technology Research Institute, Tehran, Iran; c Department of Medical Laboratory Sciences, Faculty of Allied Medicine, Tehran University of Medical Sciences, Iran; d Zoonosis Research Center, Tehran University of Medical Sciences, Tehran, Iran

**Keywords:** Vitamin A was injected intraperitoneally at 100 and 400 mg/kg two hours before 2 Gy of gamma radiation. Animals were sacrificed after 24 h, and then specimens of the bone marrow were smeared and stained. The number of micronuclei were counted in polychromatic cells [1–6], Radioprotectors, Vitamin A, Micronucleus test, Gamma radiation

## Abstract

Radioprotectors by neutralizing the effects of free radicals, reduce the destructive effects of radiation. In this protocol article, the radioprotectory effect of vitamin A on micronuclei induced by gamma radiation was evaluated using micronucleus test. Vitamin A was injected intraperitoneally at 100 and 400 mg/kg two hours before 2 Gray (Gy) of gamma radiation. Animals were sacrificed after 24 h, and then specimens of the bone marrow were smeared and stained. The number of micronuclei were counted in polychromatic cells. Both dosage of vitamin A reduced the micronucleus in bone marrow polychromatic erythrocytes (MnPCE) level, which is statistically significant. The appropriate amount of vitamin A for protection in mice is 100 mg/kg, which protect the bone marrow of mice against clastogenic effects of radiation. The results of the study showed that vitamin A, possibly with an antioxidant mechanism, eliminates the effects of free radicals from ionizing radiation on bone marrow cells and reduces genetic damage.

• The data of radioprotective effects of vitamin A showed that administration of 100 mg/kg vitamin A to mice prior to 2 Gy of gamma radiation has reduced the micronucleus levels in PCE cells by a factor of 2.62.
• Administration of 100 mg/kg vitamin A, which is much smaller than LD50 of vitamin A (LD50 for intraperitoneal injection = 1510 ± 240 mg/kg) can protect mice.
• Vitamin A reduces the harmful effects of ionizing radiation on DNA, due to the antioxidant activity and the trapping of free radicals produced by radiation, and diminish the genetic damage caused by radiation.
• Vitamin A has no effect on the proliferation and differentiation rate of bone marrow cells.

## Graphical abstract

**Figure:**
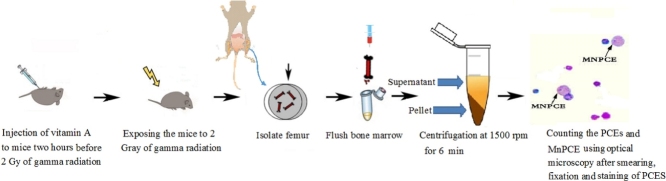

**Specifications Table**

**Table:** 

Subject area:	Medical physics
More specific subject area:	Determine the Radioprotective Effects of Vitamin A Against Gamma Radiation
Type of data:	Graph
Method name:	Vitamin A was injected intraperitoneally at 100 and 400 mg/kg two hours before 2 Gy of gamma radiation. Animals were sacrificed after 24 h, and then specimens of the bone marrow were smeared and stained. The number of micronuclei were counted in polychromatic cells [[1], [2], [3], [4], [5], [6]]
Name and reference of original method:	Radioprotective Effects of Vitamin A Against 2 Gray Gamma Radiation in Mouse Bone Marrow Cells
Resource availability:	data

Description of protocol

The radioprotective effect of Vitamin A on reducing the percentage of MnPCE in bone marrow cells are presented in Graph 1. The percentage of MnPCE in group 2 Gy Gamma radiation compared to control group increased by 74%. The difference in abundance of micronucleus in the irritated group and the control group, receiving only normal saline serum, was statistically significant (p < 0.05). Groups of mice receiving vitamin A at 100 and 400 mg/kg, two hours before irradiation, reduced the amount of micronucleus in PCE cells. Although there is a decrement in the micronucleus in PCE cells by increasing the dose of vitamin A from 100 to 400 mg/kg, the difference was statistically not significant (p > 0.05). In the control group and in the irradiated group (Graph 2), the ratio of PCE/(PCE + NCE) was not statistically significant (p > 0.05). Vitamin A in 100 and 400 mg/kg reduces the percentage of micronucleus with factor 2.62 and 2.56 compared to the group receiving only 2 Gy of gamma radiation.

**Graph 1:**
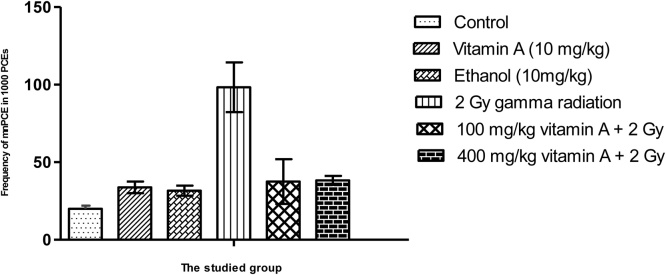
The Effect of vitamin A on the reduction of micronucleus in PCE cells with 2 Gy of gamma radiation.

**Graph 2:**
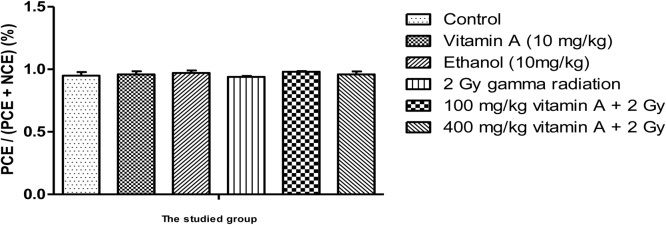
The Effect of vitamin A on the percentage of PCE/(PCE + NCE) in the bone marrow of irradiated mice with 2 Gy of gamma radiation.

## Materials and methods

### Drugs treatments

Vitamin A were supplied from DarouPakhsh Pharmaceutical Co. (Tehran, Iran). 2 h before exposure, vitamin A was injected intraperitoneally into mice.

### Animals

In this study, male NMRI mice weighing 25 ± 5 g which were purchased from Pharmacology College of Tehran University of medical sciences (Tehran, Iran). Animals were kept in the laboratory under appropriate temperature, light conditions, and standard nutrients. The mice were divided into six groups. The groups were control group, radiation group, vitamin A groups with different values (100 and 400 mg/kg) with radiation, and Vitamin A alone (Table 1). Mice were purchased at the age of 6 weeks.

**Table 1:** The animals were categorized into six groups according the following table.

Dose of gamma radiation (Gy)	Dose of Vitamin A (mg/kg)	Group
0	0	Control
2	0	Gamma radiation
0	100	Vitamin A
0	100	Ethanol 5%
2	100	Vitamin A
2	400	Vitamin A

### Irradiation

The irradiation was carried out by Varian Clinac 2100C medical linear accelerator (LINAC) for 6 MV photon in beam Linear Accelerator Center, Tehran, Iran. The source to surface distance (SSD) was 100 cm, and dose rate was 100 cGy min^−1^. The mice that were considered to be irradiated were placed in a fiberglass cage and exposed to 2 Gy of gamma radiation.

### Micronucleus test

Micronucleus test was performed according to Schmid method [7]. 24 h after irradiation, the mice were killed by cervical dislocation. Then, both femur bones were removed and contents of bone marrow were flushed out by fetal cow serum using 1 cc syringe, from the lower femoral end and collected into a micro tube. The micro tubes were centrifuged at 1500 rpm for 6 min, transferred to the slides, and the slides were fixed by methanol. Then, the slides were stained with May-Grünwald-Giemsa (Merck Company, Germany). Staining process had three steps: The fixed smears were stained in May-Grünwald dye which was diluted with an equal volume of water; placing the smears without washing into 10% Giemsa for 30 min; washing the smears in distilled water and let them to dry. For each group, 5 mice were selected, and the cells were counted with optical microscopy. For each mouse, a total of 1000 polychromatic erythrocytes were counted. Polychromatic erythrocytes and normochromatic erythrocytes were observed in blue-violet and yellow-orange, respectively.

### Statistical analysis

The mean ± SD of MnPCE/1000 PCE and (PCE/(PCE + NCE) (%)of each group was calculated. In order to select the appropriate analyzing test the distribution of data was evaluated by Shapiro–Wilk test. The data of MnPCE/1000 PCE hadn’t a normal distribution, thus, the data of MnPCE/1000 PCE were analyzed by Kruskal–Wallis test. The data of (%) PCE/(PCE + NCE) had a normal distribution, so they were analyzed by ANOVA. In the cases, which P < 0.05 the Scheffé’s method was used as post hoc test to compare the mean values of each group with other group. P < 0.05 considered as a significant value of ANOVA, Kruskal–Wallis test and Scheffé’s method.

### Ethical considerations

The Ethical Committee for medical Research at Tehran University of Medical Science, approved this research [ethical code IR.TUMS.SPH.REC.1396.4098].

## Conclusion

Vitamin A reduces the harmful effects of ionizing radiation on DNA, due to the antioxidant activity and the trapping of free radicals produced by radiation, and diminish the genetic damage caused by radiation to the bone marrow.

## Conflict of interest

The authors of this article declare that they have no conflict of interests.
